# Models with higher effective dimensions tend to produce more uncertain estimates

**DOI:** 10.1126/sciadv.abn9450

**Published:** 2022-10-19

**Authors:** Arnald Puy, Pierfrancesco Beneventano, Simon A. Levin, Samuele Lo Piano, Tommaso Portaluri, Andrea Saltelli

**Affiliations:** ^1^ School of Geography, Earth and Environmental Sciences, University of Birmingham, Birmingham B15 2TT, UK.; ^2^ Department of Ecology and Evolutionary Biology and High Meadows Environmental Institute, Guyot Hall, Princeton University, Princeton, NJ 08544-1003, USA.; ^3^ Centre for the Study of the Sciences and the Humanities (SVT), University of Bergen, Parkveien 9, PB 7805, 5020 Bergen, Norway.; ^4^ Operations Research and Financial Engineering Department, Princeton University, Princeton, NJ 08544, USA.; ^5^ University of Reading, School of the Built Environment, JJ Thompson Building, Whiteknights Campus, Reading RG6 6AF, UK.; ^6^ ETH-Zürich (ETHZ), Rämistrasse 101, 8092 Zürich, Switzerland.; ^7^ Barcelona School of Management, Pompeu Fabra University, Carrer de Balmes 132, 08008 Barcelona, Spain.

## Abstract

Mathematical models are getting increasingly detailed to better predict phenomena or gain more accurate insights into the dynamics of a system of interest, even when there are no validation or training data available. Here, we show through ANOVA and statistical theory that this practice promotes fuzzier estimates because it generally increases the model’s effective dimensions, i.e., the number of influential parameters and the weight of high-order interactions. By tracking the evolution of the effective dimensions and the output uncertainty at each model upgrade stage, modelers can better ponder whether the addition of detail truly matches the model’s purpose and the quality of the data fed into it.

## Teaser

Without validation data, more complex models may succumb to uncertainty.

## INTRODUCTION

Many mathematical models are getting increasingly complex with the assumption that ever-comprehensive representations of the process under study will eventually bound uncertainties and produce more accurate insights (*1*–*3*). This trend has been fueled by computational advances that have permitted to increase the number of transistors in a chip and thus the speed of arithmetic operations without substantially increasing power requirements—the combination of what is known as Moore’s law (*4*) and Dennard’s scaling (*5*). Such breakthroughs have allowed the simple general circulation models of the 1960s to evolve into comprehensive atmosphere-ocean general circulation models (*6*, *7*), or the “bucket”-type models of the 1970s to turn into global hydrological models that simulate water uses and the human impact on the global water cycle (*8*, *9*). In mathematical epidemiology, the basic compartmental models of Kermack and McKendrick (*10*) have expanded into the Imperial College’s transmission model of severe acute respiratory syndrome coronavirus 2 (SARS-CoV-2), based on more than 900 parameters (*11*).

In the environmental/climate sciences, hydrology, and epidemiology domains, the development of finer-grained models often proceeds without having, at the scale required, specific data available to train or validate the model (*12*). Models tend to be based on physical laws or principles specific to the field and hence may project their estimations, predictions, or dynamics into the unknown. When the lack of validation data renders the assessment of model bias (i.e., how far off are the modeled estimates from the data available) unfeasible, modelers cannot benefit from existing statistical instruments that help balance model complexity with error so as to align with science’s quest for parsimony, such as Akaike’s (*13*) or Schwarz’s (*14*) information criterion. In these cases, model expansion may proceed without standardized approaches to assess whether the level of model detail matches the quality of the knowledge available, whether the newly added processes truly lead to more accurate estimates, or how the output uncertainty builds up at each model upgrade stage. This often limits the social usefulness of process-based models as instruments to inform policies in the real world (*15*), where reliance on excessively complex and overconfident models may have deleterious social-environmental consequences (*16*).

Here, we show that modelers can gauge the connection between model complexity and uncertainty at all stages of model development by calculating the model’s “effective dimensions,” that is, the number of influential parameters and active higher-order effects (*17*–*19*). The concept of effective dimensions helps to better tune the level of model detail to the context and purpose of the application, ultimately improving the quality of models that do not (or cannot) fit any training or validation data. We also provide numerical evidence that the addition of model detail in process-based models tends to produce more (and not less) uncertain estimates because it increases the model’s effective dimensions, which generally boost the output variance. This fact, which may have gone unnoticed as yet because of the scarce uptake of uncertainty and sensitivity analyses in mathematical modeling (*20*), suggests that the quest toward ever-detailed mathematical models as a means to get sharper estimates or insights should be reassessed.

We first describe the mathematical foundations of our approach and the statistical theory behind the connection between model uncertainty, complexity, and the notion of effective dimensions. After illustrating this relation with a numerical simulation, we stress-test our theory with a meta-model that generates a very broad range of functional forms and faithfully reproduces the effect of complexification at higher effective dimensions. We then illustrate how the concept of effective dimensions can help modelers balance model complexity with uncertainty using several increasingly complex models of the energy, agriculture, and epidemiology domain. We conclude by discussing the implications of our results for the design of mathematical models and model-based policies.

## RESULTS

### Model complexity and uncertainty

We regard the number of parameters and the pattern of their connections as key contributors to the complexity of mathematical models (*21*, *22*). Our approach thus relies on the notion of “aggregate complexity” (*23*) and focuses on system components and their interactions when no validation data are available. This can be the case of predicting the evolution of a new epidemic, exploring the impact of a new technology, predicting the potential scarcity of a natural resource, and so on, over a myriad of settings often encountered in impact assessment studies.

By setting the focus on the number of model parts and their connections, we can relate model complexity with uncertainty through statistical theory via the analysis of variance (ANOVA) decomposition framework and the notion of effective dimensions (*17*–*19*) (see Materials and Methods). In this framework, parameters are regarded as stochastic variables whose uncertainty is described by probability distributions reflecting their measurement error, natural variation, inherent randomness, or the subjective judgement of experts (*24*). Given a model of the form *y* = *f*(***x***), ***x*** = (*x*_1_, *x*_2_, …, *x_i_*, …, *x_k_*) ∈ ℝ*^k^*, where *y* is a scalar output and *x*_1_, …, *x_k_* are *k* independent parameters, we can calculate the proportion of variance conveyed to *y* by each parameter (first-order effect, *S_i_*) and by the interaction between pairs of parameters (second-order effect, *S*_*i*,*j*_), triplets of parameters (third-order effect, *S*_*i*,*j*,*l*_), etc., up to the *k*th-order interaction. For a model with just three parameters, this is *S*_1_ + *S*_2_ + *S*_3_ + *S*_1,2_ + *S*_1,3_ + *S*_2,3_ + *S*_1,2,3_ = 1. This variance decomposition applies when *f*(***x***) is square-integrable over the dominion of existence, and is linked to Sobol’s (*25*) functional decomposition scheme, where *f*(***x***) is decomposed as the sum of 1,2, …, *k*-dimensional functions.

The estimation of up to the *k*th-order interaction may be impractical for high-dimensional or computationally demanding models. Under these circumstances, the calculation of the total-order effect (*T*_*i*_) allows one to capture the proportion of variance conveyed to *y* by the first-order effect of *x*_*i*_ jointly with its interactions up the *k*th order (*26*). For parameter *x*_1_ in a three-parameter model, this would be $T_{1}=S_{1}+S_{1,2}+S_{1,3}+S_{1,2.3}$ and similarly for *x*_2_ and *x*_3_. We can now introduce the concept of effective dimensions (*17*–*19*).

#### Effective dimension in the “superposition” sense

Let λ = {1,2, …, *k*}. For any subset ***u*** ⊆ λ, let ∣***u***∣ denote its cardinality (*18*). In the “superposition sense,” the effective dimension of a model *f* is the smallest integer *k_s_* such that

$$\sum_{0<|u|\leq k_{s}}S_{u}\geq p$$ (1)

where 0 < *p* < 1. The value of *p* is arbitrarily set, and here, we use *p* = 0.99 following Caflisch *et al.* (*17*). Using again a three-parameter model as an example, if (*S*_1_ + *S*_2_ + *S*_3_ + *S*_1,2_ + *S*_2,3_ + *S*_1,3_) ≥ *p*, then ∣***u***∣= *k_s_* = 2. The effective dimension *k_s_* is therefore the order of the highest effect that needs to be included in Eq. 1 to reach *p*. Models with a high effective dimension in the superposition sense have important high-order effects and hence can be considered as a sum of *k_s_*-dimensional functions (see Materials and Methods and Eq. 4) (*19*). Since *k_s_* reflects the weight of interactions between the model’s parameters, *k_s_* is also a proxy for the pattern of connections in the model. For computation simplicity, here we calculate *k_s_* up to the third-order indices and assume that *k_s_* ≥ 4 when (∑ *S_i_* + ∑ *S*_*i*, *j*_ + ∑ *S*_*i*,*j*,*l*_) < *p*.

#### Effective dimension in the “truncation” sense

Let us now consider the vector of total-order indices ***T*** = {*T*_1_, *T*_2_, …, *T_k_*}. In the truncation sense, we define the effective dimension of a model *f* to be the integer *k_t_*, such that

$$k_{t}=|C|=|\{T_{i}\in T|T_{i}>q\}|$$ (2)

where ∣***C***∣ is the cardinality of the subset ***C*** formed by the number of elements *T_i_* in ***T*** such that *T_i_* > *q*. Here, we define *q* = 0.05 as this is the threshold commonly used in sensitivity analysis to screen “influential” from “noninfluential” parameters (those that convey uncertainty to *y* from those that do not). Models with a high effective dimension in the truncation sense have a large number of influential parameters and thus a large *k_t_* value. Note that our definition of effective dimension in the truncation sense is a simplification of the notion introduced by Wang and Fang (*18*) and Caflisch *et al.* (*17*). Here, we give precise *k_t_* values for all the models examined.

**Fig. 1.**
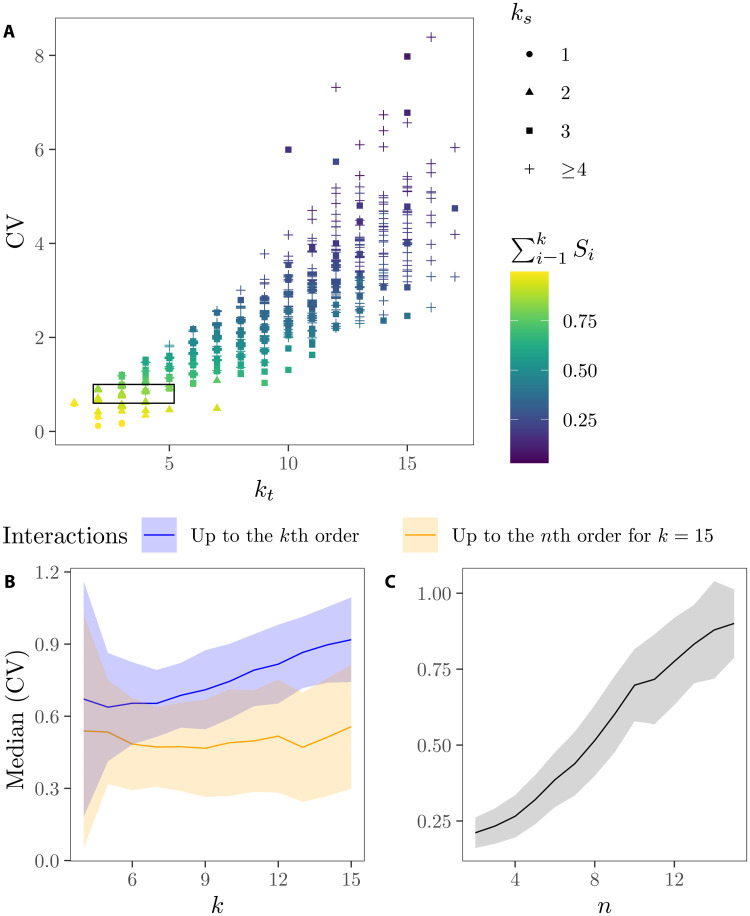
The link between model complexity and uncertainty. (**A**) Trend between the effective dimension in the truncation sense (*k_t_*), the effective dimension in the superposition sense (*k_s_*), the sum of the first-order effects of the models’ parameters *S_i_*, and the coefficient of variation (CV), which we use as a proxy for uncertainty. Each dot is a simulation (*N* = 2^10^) that randomizes the coefficients of Sobol’s G function (*68*) (see Materials and Methods). The small rectangle frames some simulations in which models with different *k_t_* and *k_s_* dimensions have an almost identical output variance. (**B**) The meta-model approach. Evolution of uncertainty through different dimensions *k* as a function of the order of the highest interaction activated, for *n* ≤ *k*. The line shows the median value, whereas the ribbon displays the SD. *N* = 2^11^. (**C**) Evolution of uncertainty after fixing the model dimensionality at *k* = 15 and activating up to the *n*th-order interaction.

Usually, *k* ≫ *k_t_* ≫ *k_s_* due to the general dominance of low-order effects in mathematical models and the preeminence of the Pareto principle (c. 80% of the effects are conveyed by c. 20% of the parameters) (*27*–*29*). Models live in the space set by *k_t_* and *k_s_* and not in that nominally defined by *k*, which may be artificially large if the model includes a non-negligible number of noninfluential parameters. The space defined by *k_t_* and *k_s_* cannot be simplified without modifying the model’s behavior, and thus, it is irreducibly complex (*30*). It follows that more complex models will generally display a higher effective dimension in *k_t_* and *k_s_*, an increase that promotes a larger output uncertainty (Fig. 1A). This is because the output variance of more complex models is increasingly driven by higher-order effects activated by the progressive addition of influential model parameters: Note in Fig. 1A how the larger the *k_s_* dimension is, the smaller the sum of first-order indices *S_i_*.

### The meta-model check

To check whether the relation between higher effective dimensions, the addition of model detail, and larger output uncertainty holds regardless of the model functional form, we stress-test our approach with a meta-model based on the Becker (*29*) metafunction. Our meta-model randomizes over 13 different univariate equations representing common responses observed in physical systems, from linear to multimodal (fig. S1) (*31*). It also randomly activates up to the *n*th-order effect, for *n* ≤ *k*, to observe how different combinations of *k* and *k_s_* values affect the output uncertainty. This approach allows the creation of more than two million models with different dimensionalities, functional forms, and degree of interactions (see Materials and Methods).

Also in this case, the output uncertainty tends to increase with the number of model parameters and the activation of up to the *k*th-order interaction (the blue trend in Fig. 1B). Note how a model with more parameters does not necessarily lead to a larger uncertainty if *n* ≠ *k* (the orange trend in Fig. 1B). This highlights the importance of high-order interactions in defining both the complexity of the model and its output uncertainty. The number of parameters can be fixed and the uncertainty increases all the same just by progressively adding new interactions between the parameters (Fig. 1C).

The width of the trend in Fig. 1 indicates that the addition of new parameters does not mechanically have to promote more uncertain estimates: Several models with different effective dimensions in both *k_t_* and *k_s_* may display the same output variance. By routinely assessing the model’s effective dimensions in every model upgrade stage, modelers can appraise the link between complexity and uncertainty during model development and ponder whether the addition of detail truly suits the model’s purpose. Optimal complexity would depend on whether the model aims at predicting variables, understanding dynamics, managing resources, or guiding policy-making. In the next section, we illustrate the practical implications of our approach using several increasingly complex models as examples.

### The link between effective dimensions and uncertainty in action

#### Compartmental models

In many fields, model complexification involves linking models in a causal chain, where the output of a given link is used as the input of the next one. In climate change research, greenhouse gas emission scenarios are usually transferred into impact models whose output is used to assess local impacts and, lastly, to design adaptation responses (*32*, *33*). In flood management, flood wave forecasts require runoff predictions, which previously demand rainfall forecasts (*34*). Basic epidemiological models also follow this layout, as individuals flow from one compartment to another depending on whether they are susceptible, infected by the virus, or recovered (*35*). Eventually, new compartments are added to improve the model’s descriptive capacity and its usefulness for policy-making.

**Fig. 2.**
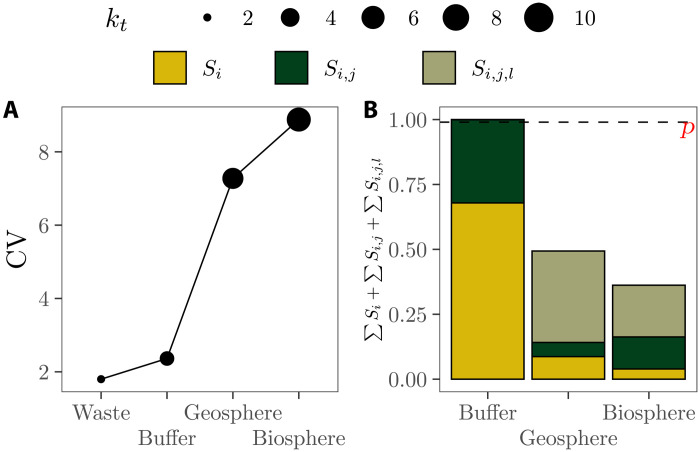
Relation between uncertainty (represented by the CV) and complexity in the PSACOIN Level 0 model. (**A**) Evolution of the CV and *k_t_* across the four compartments of the PSACOIN Level 0 model. (**B**) Proportion of the model output variance explained by the sum of first (*S_i_*)–, second (*S*_*i*,*j*_)–, and third (*S*_*i*,*j*,*l*_)–order effects. The horizontal dashed line is at *p* = 0.99. Note that the waste level is missing because it only contains one uncertain parameter and thus *k_t_* = *k_s_* = 1.

We illustrate how adding complexity affects the effective dimensions of compartmental models with the Probabilistic System Assessment Code Intercomparison (PSACOIN) Level 0 model (*36*). It simulates the leaking of radionuclides from a given repository and its transport to a buffer, then to the geosphere, and lastly to the biosphere, where they are ingested by humans and animals after drinking water from a contaminated well or stream (figs. S2A and S3). Overall, the PSACOIN Level 0 model includes 10 uncertain parameters described with probability distributions and 11 constants (table S1). If the model’s effective dimensions are assessed in every model upgrade stage, the progressive rise in *k_t_* and *k_s_* unfolds: *k_t_* goes from *k_t_* = 1 in the waste level to *k_t_* = 2, *k_t_* = 5, and *k_t_* = 6 in the buffer, geosphere, and biosphere level, respectively (Fig. 2A). As for *k_s_*, it goes from *k_s_* = 2 in the buffer level to *k_s_* ≥ 4 in the geosphere and biosphere level, since the sum of up to third-order effects in these levels is not enough to reach *p* (Fig. 2B). Note how uncertainties get larger in each model complexification stage, with the largest increase taking place in the transition between the buffer and the geosphere model: This is the critical leap in the development of the PSACOIN Level 0 model in terms of output uncertainty and complexity.

Flagging the progression of *k_t_* and *k_s_* values and related uncertainty in each PSACOIN upgrade stage allows one to see that the regulation on the safety of nuclear waste disposal should not set limits on doses to humans in the biosphere [as is usually done in the United States; see the case of the Yucca Mountain nuclear waste repository in Nevada (*16*, *37*, *38*)]. This is because once the contaminant reaches the buffer-geosphere interface, its fate is extremely hard to predict, and the addition of model detail just boosts the uncertainty. Regulations would thus establish more realistic and defensible safety standards if they define a maximum level of radioactivity leaving the buffer onto the aquifer and the geosphere (expressed in becquerels per year) rather than a total dose to the biosphere (expressed in sieverts per year). The PSACOIN Level 0 case illustrates how the concept of effective dimensions may help regulators distinguish in compartmental models which compartment produces the most solid inferences to guide policy-making, and where is the threshold beyond which the addition of complexity no longer makes the model fit for purpose.

#### Structural uncertainties

During the upgrade of a model, modelers may be unsure as to which is the best way to mathematically represent the newly added process. This structural uncertainty, also known as process-based uncertainty (*39*), often needs to be appraised to discern how the selection of a given equation over another conditions the model’s behavior and its output. The link between model complexity and uncertainty can be examined to unfold the uncertainty buildup due to the accumulation of both parametric and structural uncertainties, and to check whether model complexification should proceed without the need to address this source of structural uncertainties.

We illustrate this exercise with a basic formula to estimate the total amount of water withdrawn to irrigate a crop. Such a formula requires the calculation of the evapotranspiration of a reference crop (usually grass or alfalfa), the calculation of the crop evapotranspiration (wheat in this case), and lastly the estimation of the water withdrawn for irrigation (*40*). However, there are c. 50 different equations available to calculate the reference evapotranspiration and no agreement as to which method might work best (*41*). Some of the most used are the Priestley-Taylor and the FAO-56 Penman-Monteith (*42*), known to provide results that can differ by a factor of 2 or more (*40*). We examine the impact that this source of uncertainty has on the model’s effective dimensions and uncertainty with a trigger (*43*), a parameter that randomly decides which evapotranspiration equation should be used in each model simulation (see the Supplementary Materials).

**Fig. 3.**
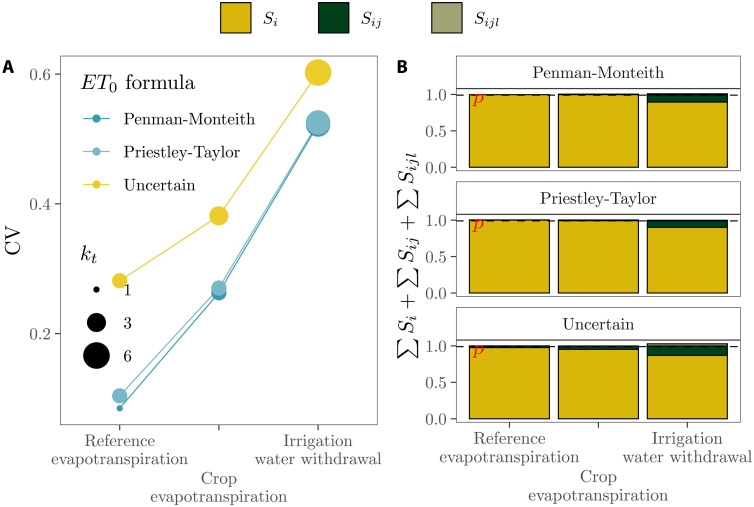
Relation between uncertainty (both parametric and structural) and complexity using the modeling of irrigation water withdrawals as a case study. (**A**) Evolution of uncertainty and *k_t_* in the three compartments needed in the model: reference evapotranspiration, crop evapotranspiration, and water withdrawal. (**B**) Sum of first-, second-, and third-order effects in each compartment as a function of the reference evapotranspiration equation (*ET*_0_) used (Penman-Monteith, Priestley-Taylor, or uncertain).

The uncertainty in the selection of the reference evapotranspiration equation slightly increases the model’s effective dimensions in all compartments: *k_t_* values rise from 2 to 3 in the crop evapotranspiration compartment and from 5 to 6 in the irrigation water withdrawal compartment (Fig. 3A). The raise in *k_s_* is also moderate: *k_s_* values increase from 1, 1, and 2 to 2, 2, and 3 in the reference evapotranspiration, crop evapotranspiration, and water withdrawal compartment, respectively (Fig. 3B). Such minor increase in complexity, however, leads to a much larger output uncertainty than the uncertainty yielded by a model that uses either the Priestley-Taylor or the Penman-Monteith equations (blue lines in Fig. 3A).

The evolution of the model complexity and output uncertainty in these irrigation models suggests that (i) opting for either the Penman-Monteith or the Priestley-Taylor equation leads to overlook an important source of structural uncertainty whose effect in the model lasts until the very last compartment, (ii) the selection of just one reference evapotranspiration equation is hard to justify on the basis of parsimony since the effective dimensions of the resulting models are similar to those of the “uncertain” model, and (iii) regardless of the model selected, the largest boost in output uncertainty takes place in the transition to the irrigation water withdrawal compartment, which also displays comparatively higher *k_s_* values. Policy-makers using irrigation models to guide action in the field should plan with regard to an uncertainty that may be irreducible given the importance of higher-order interactions.

#### Complexification of compartmental models

The addition of model detail often goes along with the establishment of new compartment flows, links, and information delays. The upgraded model is then no longer a causal chain but a system with feedback loops and several input-output relationships. This is common practice in epidemiology, where the descriptive capacity of the classic susceptible-infected-recovered (SIR) model is often enhanced with the addition of fine-grained features such as seasonality or age stratification (*44*).

Such complexification may also produce fuzzier estimates at each model upgrade stage due to the increase in the model’s effective dimensions. To illustrate this fact, we propagate uncertainties in three increasingly detailed SIR-based models, extracted from Saad-Roy *et al.* (*45*, *46*): a SIR-based model with waning immunity and no vaccination [SIR(S)], a SIR upgraded with a vaccination compartment [SIR(S-V)], and an extended SIR with different vaccination strategies [SIR(S-E)] (figs. S2, B to D, and S4). These SIR models aimed at providing insights into the magnitude of future SARS-CoV-2 cases in 2020-2021 given different assumptions on the nature of the adaptive immune response and pharmaceutical interventions.

**Fig. 4.**
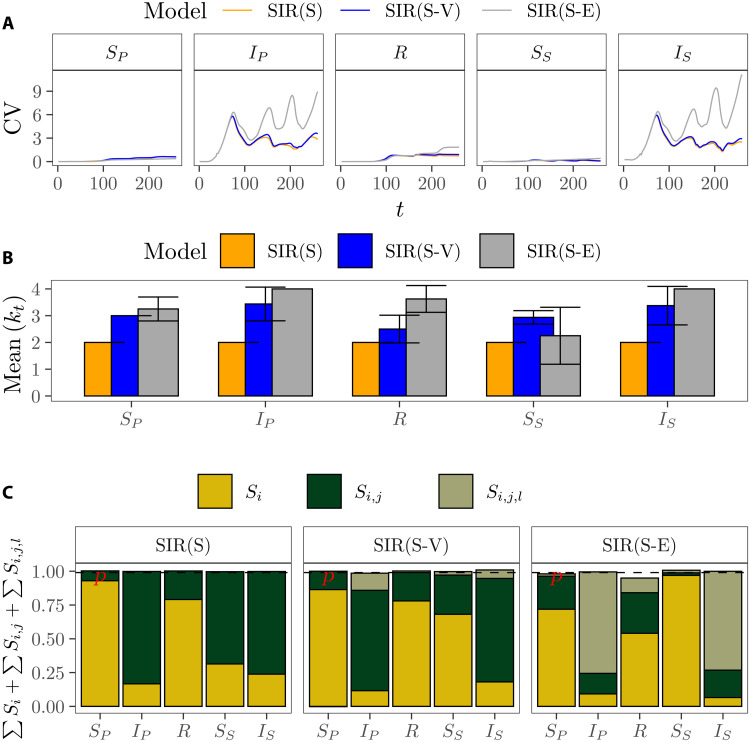
Relation between complexity and uncertainty in three SIR(S)-based models. Models from Saad-Roy *et al*. (*45*, *46*). (**A**) Evolution of the uncertainty across time in the five compartments shared by all the models: fully susceptible individuals (*S_P_*), fully immune individuals as a result of recovery from either primary or secondary infection (*I_P_*), individuals that have recovered (*R*), individuals whose immunity has waned (*S_S_*), and individuals with secondary infection that transmit at reduced rate (*I_S_*). (**B**) Mean *k_t_* values of each of the compartments for each of the SIR(S) models considered. The error bar shows the SD. (**C**) Proportion of the model output uncertainty at *t* = 200 explained by the sum of first-, second-, and third-order effects. The horizontal dashed line is at *p* = 0.99.

Figure 4A indicates that the simplest SIR, the SIR(S), generally provides the least uncertain estimates, followed closely by the SIR(S-V). The more detailed model, the SIR(S-E), yields the most uncertain estimates, especially for *I_P_* and *I_S_* at *t* > 80. The progressive increase in uncertainty as a result of the addition of model detail is mirrored by a gradual raise in the effective dimensions: Notice how *k_s_* = 2 in all compartments of the SIR(S), *k_s_* = 3 in all compartments of the SIR(S-V), and *k_s_* ≥ 4 for the compartments *S_P_*, *I_P_*, *R*, and *I_S_* of the SIR(S-E), since at least fourth-order effects are needed to reach *p* (Fig. 4, B and C).

**Fig. 5.**
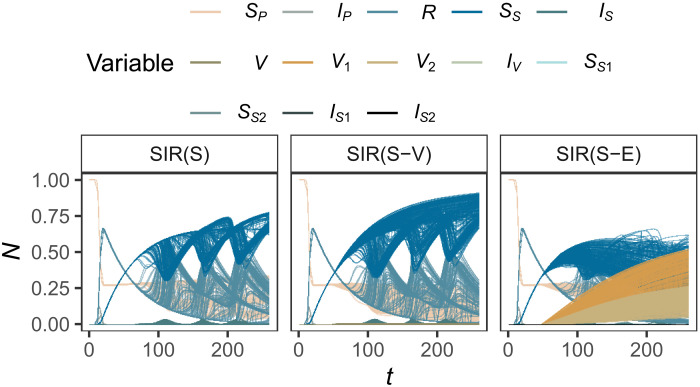
Monte Carlo propagation of uncertainties in three SIR-based models. Models from Saad-Roy *et al.* (*45*, *46*). Each of the 128 lines in each state variable and model reflects a simulation conducted with parameters taking specific values according to their uncertainty range. *V*, vaccinated; *V*_1_, one-dose vaccinal immunity; *V*_2_, two-dose vaccinal immunity; *I_V_*, infection after vaccination; *S*_*S*_1__, waned one-dose immunity; *S*_*S*_2__, waned two-dose immunity; *I*_*S*_1__, infection after waned one-dose immunity; *I*_*S*_2__, infection after waned two-dose immunity. For the rest of the state variables, see the caption of Fig. 4.

The link between the output variance and the concept of effective dimensions permits to ponder which SIR model offers more appropriate insights to guide action. Note how the SIR(S) and the SIR(S-V) display an almost identical output variance through all the time intervals considered (Fig. 4A), and yet the SIR(S-V) has higher *k_t_* and *k_s_* values (Fig. 4, B and C). The propagation of uncertainty also evidences that the increase in the effective dimensions in the upgrade from the SIR(S-V) to SIR(S-E) blurs the evolution of susceptible, infected, and recovered individuals; the relative effects of vaccination programs; or the best timing for interventions from *t* > 80 onward (Fig. 5). If the goal of the model is to gain insights into the effects that vaccination and nonpharmaceutical interventions may have on the spread of the virus, then the SIR(S-V) might be preferred over the more complex SIR(S-E) because the extra detail in the latter does not help clarify potential courses of action. However, if the goal of the SIR-based model is to inform on the potential evolution of infected and susceptible individuals, then the SIR(S) might be favored over the SIR(S-V) because the higher effective dimensions of the latter do not provide any significant additional insights into the possible dynamics of the epidemic to justify its higher detail (Fig. 5).

## DISCUSSION

Here, we analyze the relation between complexity and uncertainty in process-based mathematical models that do not (or cannot) rely on a training and/or validation dataset. Such models, ubiquitous and often prevalent at the science-policy interface, provide insights into complex issues that may escape verification: How many radionuclides can leak from a waste repository and get into humans via drinking water? How will a new pandemic spread? How much water for irrigation will be needed worldwide by 2050? Will the detonation of the first nuclear device set fire to the atmosphere? What is the cost of CO_2_ averaged over the next century? For these types of questions, we show that the addition of model detail promotes fuzzier estimates because it generally increases the model’s effective dimensions *k_t_* (the number of influential parameters) and *k_s_* (the order of the highest-order effect active in the model). This makes the model’s uncertain space more dominated by interactions, which promote larger uncertainties. The addition of complexity, rather than sharpen the accuracy of the estimation, may swamp it under indeterminacy.

Greater complexity can be useful if the scope of the mathematical model is to explore the implications of its underpinning assumptions and theories. A higher level of detail permits the modeled process to exhibit in full its range of variation once the hyperspace of its input parameters is thoroughly explored, an insight that may help detect hitherto unexpected behaviors. In other words, “model responsibly” rather than “simple is beautiful” is the stance of the present work. What is undesirable is the coupling of excess complexity with the illusory accuracy that results from scarce attention to the propagation of uncertainties onto the prediction of the model. This practice may miss on the unanticipated behaviors, foster tunnel vision, and underestimate serious socioenvironmental risks such as the effects of climate change (*47*), the impact of insecticides on bees (*48*) or the environmental impact of irrigated agriculture (*40*, *43*). Among practitioners, this pathology is known as “garbage in, garbage out” (“where uncertainties in inputs must be suppressed lest outputs become indeterminate”) (*49*).

Examining the link between effective dimensions and output uncertainty at each model upgrade stage may improve the quality of process-based models. The lack of validation data at the spatial/temporal scales required means that these models often only have access to the measurement error part of the systemic bias and measurement error trade-off, known as the O’Neill (*50*) conjecture in ecology, as Zadeh’s (*51*) principle of incompatibility in systems analysis, or as the “bias-variance trade-off” in machine learning (*52*). The concept of effective dimensions, however, enhances the examination of measurement error by tracking the uncertainty buildup at each model upgrade stage and facilitating consideration of a broad set of quality criteria beyond goodness-of-fit statistics, such as model purpose, complexity, and transparency. It sets the ground for a reflective (and not mechanical) approach to model selection, along the lines of what Fisher and Neyman recommended as best practices in inferential statistics (*53*). Unlike information criterion, the calculation of the effective dimensions can also integrate epistemic uncertainties [uncertainty due to the imperfection of our knowledge (*54*)], which often expand when models get enlarged to include new processes. This exercise ensures that model complexity remains within bounds compatible with the quality of the evidence fed into the model itself, ultimately providing modelers with insights to help them select the “most appropriate” model.

Our approach is especially valuable for models that aim at playing a role in policy arenas. Pinpointing the model’s effective dimensions can contribute to take more robust actions because the space of possible options is no longer artificially constrained by spuriously accurate model outputs. Both modelers and their constituency gain by a process of transparent communication of the uncertainty, which results from proper monitoring of each model upgrade stage. This facilitates the identification of the “stopping point” beyond which the addition of extra detail no longer makes the model suitable, thus promoting a reciprocal domestication between models and society (*15*). Such a control is especially needed to prevent policy-oriented models from getting as complex as to cloak value-laden assumptions and output uncertainties (*55*). For instance, how many readers outside the circle of practitioners know that the 1.5 degree climate target is achieved in integrated assessment models thanks to the adoption of so-called negative emissions, a technology still to be fully developed (*56*)? Or that global hydrological models assume that irrigation optimizes crop production and water use (a premise at odds with the practices of traditional irrigators) (*40*)?

Since larger-scale models can command more epistemic authority (*57*), modelers may be tempted to add detail to reinforce their status as influential actors at the science-policy interface and gain recognition and reward (*58*). More complex models are also more ductile to political calibration, a sequential process of continuously refining the fit between modeling and policy requirements (*56*). In this situation, the model becomes a moving target as it is continuously rearranged to include new data and processes to match evolving policy needs, with no evidence as to how it works prospectively. This makes larger models more prone to fall into the “Texas sharpshooter” fallacy, where you shoot first and then draw the bull’s-eye where the bullet hits. The calculation of the model’s effective dimensions may contribute to prevent or minimize these biases by systematically unfolding the balance between model prowess, uncertainty, and policy goals.

Our work also stresses the importance of stripping mathematical models of superfluous parameters, processes, or linkages. This is related to Occam’s razor principle by which “entities should not be multiplied without necessity” (*59*). There are few reasons to keep models in their nominal dimension *k* when *k* ≫ *k_t_*, because such inequality points to unneeded redundancy. An example is the irrigation module of many global hydrological models (*60*, *61*): Although computationally demanding and formed by several parameters and equations, their behavior is ultimately very similar to a linear regression of irrigated areas (*62*). This suggests the existence of a mismatch between their “real” complexity and their computational requirements. The concept of effective dimensions, by distilling the model from superfluous elements, refines also the notion of algorithmic complexity down to its “true” value (*23*).

The key dimension determining the model output uncertainty is *k_s_*, i.e., the order of the highest-order effect active in the model. This dimension cannot be pinpointed by intuition alone and is often computationally elusive if *k* is high. However, the influence of high-order effects can be pondered with the calculation of the total-order index *T_i_*. This requires submitting the model to a global sensitivity analysis (*63*). If many inputs are uncertain and the model is time-consuming to run, this may prove impossible: A model that takes days to run for a single combination of its inputs cannot be run hundreds, let alone thousands of times, to properly characterize its uncertainty space (*64*). The trend toward increasingly complex process-based mathematical models discussed in Introduction may be grounded in this paradox: More detailed models may be thought of as more accurate simply because their very design complicates any attempt at proving otherwise.

## MATERIALS AND METHODS

### Variance-decomposition framework

Given a model of the form *y* = *f*(***x***), ***x*** = (*x*_1_, *x*_2_, …, *x_i_*, …, *x_k_*) ∈ ℝ*^k^*, where *y* is a scalar output and *x*_1_, …, *x_k_* are *k* independent parameters whose uncertainty is described by probability distributions, the variance of the output *V*(*y*) can be decomposed as

$$V(y)=\sum_{i=1}^{k}V_{i}+\sum_{i}\sum_{j>i}V_{i,j}+\ldots +V_{1,2,\ldots ,k}$$ (3)

where *V_i_*, *V*_*i*,*j*_, … are, respectively, the conditional variances of *x_i_*, (*x_i_*, *x_j_*), … on *y*. Equation 3 is linked to Sobol’s (*25*) functional decomposition scheme

$$\begin{array}{cc} f(x) & =f_{0}+\sum_{i=1}^{k}f_{i}(x_{i})\\ & +\sum_{i}\sum_{j>i}f_{i,j}(x_{i},x_{j})+\ldots +f_{1,2,\ldots ,k}(x_{1},x_{2},\ldots ,x_{k}) \end{array}$$ (4)

given that

$$\begin{array}{c} f_{0}=E(y)\\ f_{i}=E_{x_{{∼}_{i}}}(y|x_{i})-E(y)\\ f_{i,j}=E_{x_{{∼}_{i,j}}}(y|x_{i},x_{j})-f_{i}-f_{j}-E(y) \end{array}$$ (5)

where *E*(.) is the mean operator and ***x***_∼*_i_*_ denotes all parameters except the *i*th, and therefore

$$\begin{array}{cc} V_{i} & =V[f_{i}(x_{i})]=V_{x_{i}}[E_{x_{∼i}}(y|x_{i})]\\ V_{i,j} & =V[f_{i,j}(x_{i},x_{j})]=V_{x_{i},x_{j}}[E_{x_{∼i,j}}(y|x_{i},x_{j})]\\ & -V_{x_{i}}[E_{x_{∼i}}(y|x_{i})]\\ & -V_{x_{j}}[E_{x_{∼j}}(y|x_{j})] \end{array}$$ (6)

Sobol’s (*25*) indices are then calculated as

$$S_{i}=\frac{V_{i}}{V(y)},S_{i,j}=\frac{V_{i,j}}{V(y)}\ldots$$ (7)

where *S_i_* is the first-order effect of *x_i_*, *S*_*i*,*j*_ is the second-order effect of (*x_i_*, *x_j_*), etc.

The total-order index (*T_i_*) captures the proportion of variance in *y* propagated by the first-order effect of *x_i_* jointly with its interactions up to the *k*th order (*26*). In other words, *T_i_* includes all terms in Eq. 3 with the index *i* and is computed as

$$T_{i}=1-\frac{V_{x_{∼i}}[E_{x_{i}}(y|x_{∼i})]}{V(y)}=\frac{E_{x_{∼i}}[V_{x_{i}}(y|x_{∼i})]}{V(y)}$$ (8)

### Uncertainty and sensitivity analysis

To assess how the addition of model complexity increases the output uncertainty, we submit all models and functions studied here to global uncertainty and sensitivity analysis. For each model, we construct a (*N*, *k*) ***Q*** matrix, where *k* is the number of model parameters and *N* is the row dimension, using Sobol’s quasi-random numbers (*65*). We allocate the leftmost *k* columns to an ***A*** matrix and the rightmost *k* columns to a ***B*** matrix. In these matrices, each column is a parameter and each row is a sampling point. Any sampling point in ***A*** or ***B*** can be indicated as *x_vi_*, where *v* indexes the row (from 1 to *N*) and *i* indexes the column (from 1 to *k*). We also construct *k* $A_{B}^{\left(i\right)}$ ($B_{A}^{\left(i\right)}$) matrices, where all parameters come from the ***A*** (***B***) matrix except the *i*th, which comes from the ***B*** (***A***) matrix.

The model *f* runs row-wise in each matrix, and we use the model output *y* to compute the coefficient of variance and the sensitivity indices. We compute *S_i_* and *T_i_* with the Azzini estimators (*66*)

$$S_{i}=\frac{2\sum_{v=1}^{N}(f{\left(B_{A}^{\left(i\right)}\right)}_{v}-f{\left(B\right)}_{v})(f{\left(A\right)}_{v}-f{\left(A_{B}^{\left(i\right)}\right)}_{v})}{\sum_{v=1}^{N}[{\left(f{\left(A\right)}_{v}-f{\left(B\right)}_{v}\right)}^{2}+{\left(f{\left(B_{A}^{\left(i\right)}\right)}_{v}-f{\left(A_{B}^{\left(i\right)}\right)}_{v}\right)}^{2}]}$$ (9)

$$T_{i}=\frac{\sum_{v=1}^{N}{\left[f{\left(B\right)}_{v}-f{\left(B_{A}^{\left(i\right)}\right)}_{v}\right]}^{2}+{\left[f{\left(A\right)}_{v}-f{\left(A_{B}^{\left(i\right)}\right)}_{v}\right]}^{2}}{\sum_{v=1}^{N}{\left[f{\left(A\right)}_{v}-f{\left(B\right)}_{v}\right]}^{2}+{\left[f{\left(B_{A}^{\left(i\right)}\right)}_{v}-f{\left(A_{B}^{\left(i\right)}\right)}_{v}\right]}^{2}}$$ (10)

We conduct all of the uncertainty and sensitivity analyses with the sensobol package in R (*67*). We provide a detailed description of the models and of the probability distributions used to characterize parametric uncertainties in the Supplementary Materials.

### Sobol’s G function

To create Fig. 1A, we randomize in a Monte Carlo setting the coefficients of Sobol’s G function (*68*), an analytically tractable, well-known function among modelers. It reads as

$$y=\prod_{i=1}^{k}\frac{|4x_{i}-2|+a_{i}}{1+a_{i}}$$ (11)

where *x_i_* ∼ 𝒰(0,1), *a_i_* ∈ ℝ^+^, *i* = 1,2, …, *k*. The typology of the function is driven by *k* and the value of the coefficients *a_i_*: If more than one parameter has a low *a*, then high-order interactions will be present. The more parameters with a low *a*, the stronger the interactions and the higher the *k_s_* dimension of the function. Here, we sample with replacement the coefficients from the set {0,1,4.5,9,99}, weighted as {0.4,0.3,0.2,0.05,0.05} to promote the creation of nonadditive functions. This approach allows one to explore the connection between *k*, *k_t_*, *k_s_*, and output uncertainty in a wide range of model settings and across models with very different behaviors.

### The meta-model

Our meta-model is based on the Becker (*29*) metafunction. Let ***u*** = {*u*_1_, *u*_2_, …, *u_k_*} be a *k*-length vector formed by randomly sampling integer values from 1 to 13, in which each integer value is linked to 1 of the 13 univariate functions in fig. S1. We then apply the *i*th function in ***u*** to the *i*th model input: If *k* = 3 and ***u*** = {*u*_3_, *u*_1_, *u*_12_}, then $f_{3}(x_{1})=\frac{\left(e^{x_{1}}-1\right)}{e-1}$, $f_{1}(x_{2})=x_{2}^{3}$, and $f_{12}(x_{3})=x_{3}^{2}$.

We also randomly activate up to the *n*th-order interaction, for *n* ≤ *k*, and create *n* matrices ***V***, …, ***W*** of dimension *k*_2_ × 2, …, *k_n_* × *n*, where $k_{2}=\frac{k!}{2!(k-2)},\ldots ,k_{n}=\frac{k!}{n!(k-n)}$. Each row in these matrices represents an *n*th-order interaction between the model inputs. Overall, this model can be formalized as

$$\begin{array}{cc} y= & \sum_{i=1}^{k}f^{u_{i}}(x_{i})+\\ & \sum_{i=1}^{k_{2}}f^{u_{V_{i,1}}}(x_{V_{i,1}})f^{u_{V_{i,2}}}(x_{V_{i,2}})\\ & +\cdots \\ & +\sum_{i=1}^{k_{n}}f^{u_{W_{i,1}}}(x_{W_{i,1}})\ldots f^{u_{W_{i,n}}}(x_{W_{i,n}}) \end{array}$$ (12)

where the first term represents the first-order effects, the second term the second-order effects, and so on up to the *n*th-order effect.

## Supplementary Materials

### This PDF file includes:

Figs. S1 to S4 The Models Tables S1 to S5 References
