# Supplementary material for: Models with higher effective dimensions tend to produce more uncertain estimates
Source: Sci Adv. 2022 Oct 19;8(42):eabn9450. doi: 10.1126/sciadv.abn9450 (PMC9581491; doi:10.1126/sciadv.abn9450)
Supplement: Supplementary file 1 — Figs. S1 to S4 The Models Tables S1 to S5 References [file sciadv.abn9450_sm.pdf]

Science Advances
AAAS

Supplementary Materials for

Models with higher effective dimensions tend to produce more uncertain estimates

Arnald Puy *et al.*

Corresponding author: Arnald Puy, a.puy@bham.ac.uk

Sci. Adv. **8**, eabn9450 (2022)
DOI: 10.1126/sciadv.abn9450

This PDF file includes:

Figs. S1 to S4
The Models
Tables S1 to S5
References

1 Figures

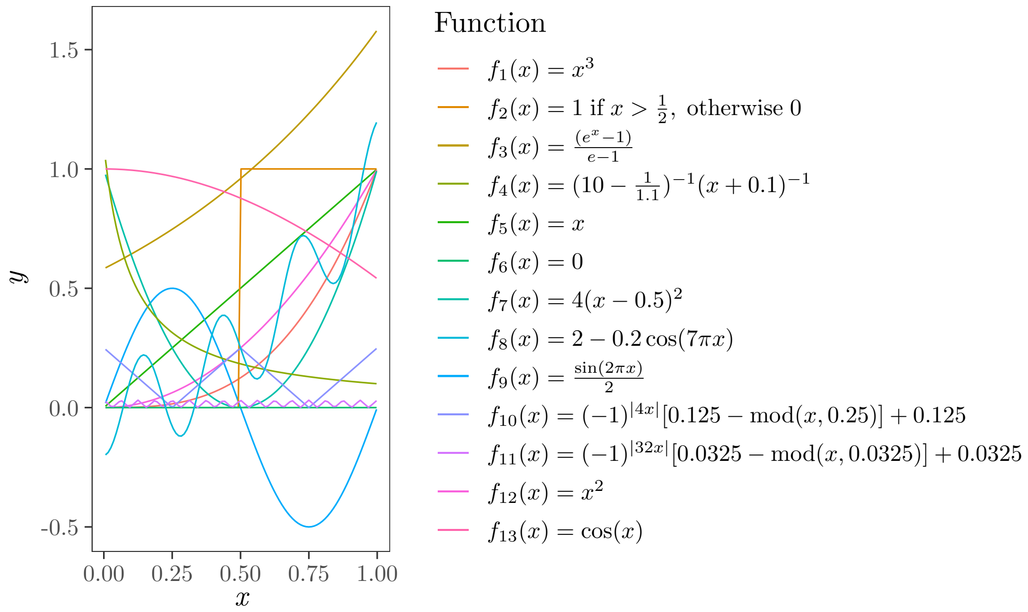

Figure S1: Univariate functions implemented in the meta-model based on the Becker [29] metafunction.

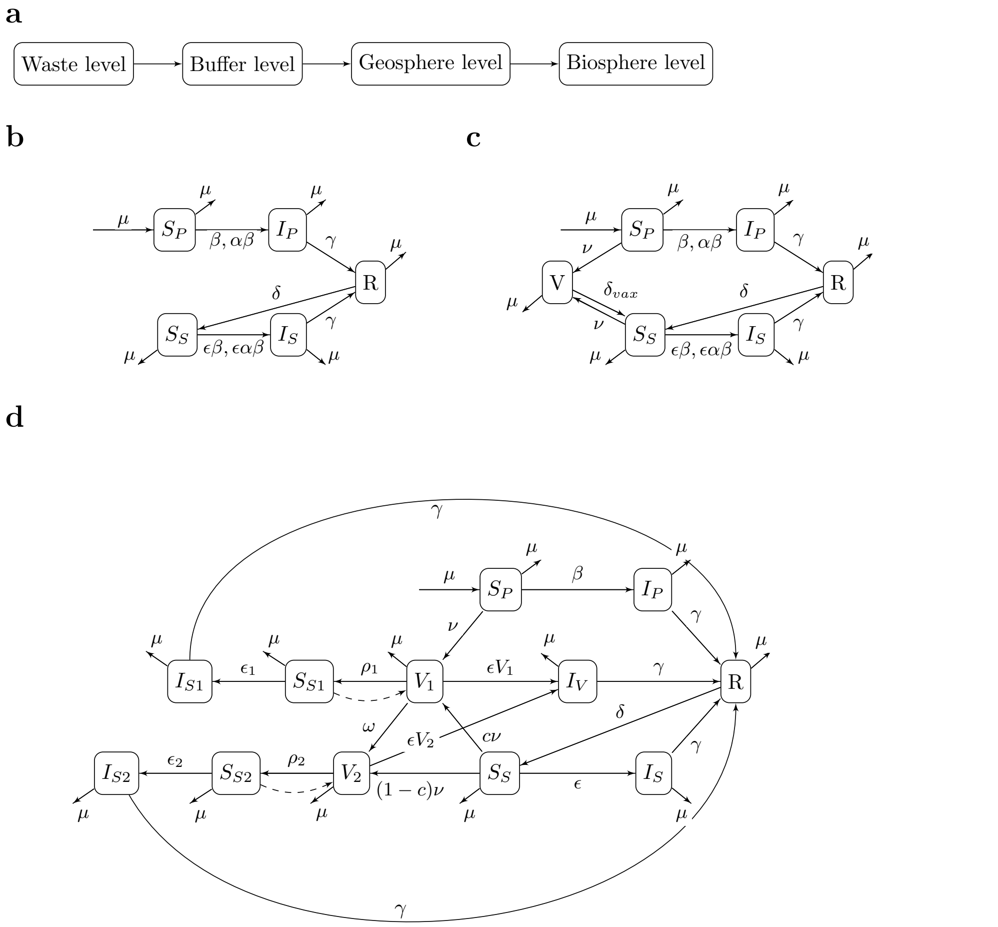

Figure S2: Diagram flows. a) PSACOIN Level 0 model [36]. b) SIR(S) model, based on Fig. 1 in Saad-Roy [45]. c) SIR(S) with a vaccinated compartment, based on Fig. 3a in Saad-Roy et al. [45]. d) Fully extended SIR(S), based on Fig 1a in Saad-Roy et al. [46].

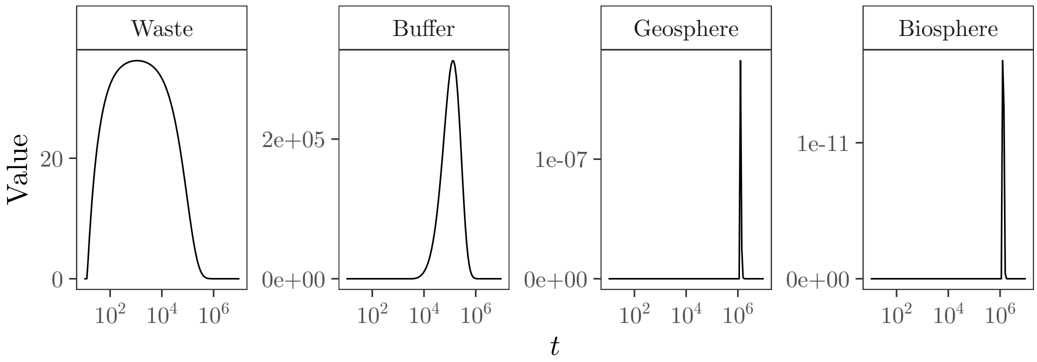

Figure S3: Dynamics of the PSACOIN Level 0 model [36]. The time t is in years.

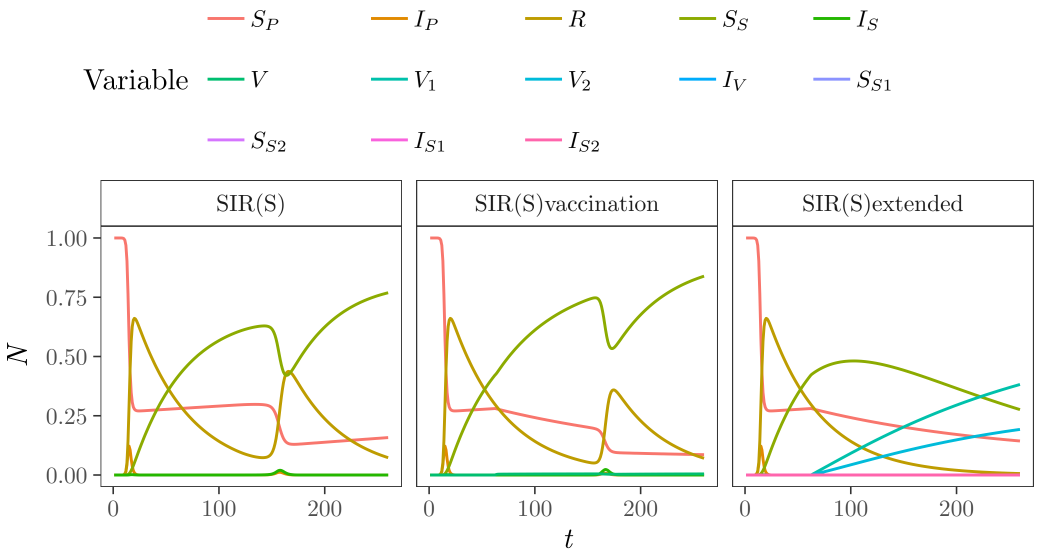

Figure S4: Dynamics of the SIR(S), the SIR(S) with vaccination and the extended SIR(S) proposed by Saad-Roy et al. [45, 46]. The time t is in weeks and covers 5 years (260 weeks). See section 2.3 for a description of the models.

2 The models

2.1 The PSACOIN model

It describes the ideal behavior of a set of selected radionuclides buried deep into an underground disposal for nuclear waste, packed into sealed canisters and surrounded by a buffer material conceived to delay their transit time in case of canister corrosion. The nuclides are separated by the biosphere by several hundred metres of a geological formation. The simulations normally span ten million years, a time supposed to be characteristic of the nuclide transit time through the various media (barriers). The case is part of a series of benchmarks runs by the Nuclear Energy Agency of the OECD, aimed to test the agreement among several computer codes involved in the analysis of the safety of nuclear waste disposal. The description that follows is a summary of the first and simplest case, PSACOIN Level 0 [37].

2.1.1 First barrier: waste form

The leach rate $R_{wf}(t)$ ($\frac{Kg}{m^2 a}$) is given by

$$R_{wf}(t) = R^0 H(t - \tau^D)\,, \tag{S1}$$

where wf stands for waste form, R^0 is a time invariant leach rate, t is time, τ^D is a characteristic leach time and H is the Heaviside step function

$$H(y) = \begin{cases} 0 & y < 0 \\ 1 & \text{otherwise} \end{cases} \tag{S2}$$

The leach rate τ^D is given by

$$\tau^D = \frac{Q}{R^0 S}\,, \tag{S3}$$

where Q is the initial amount of waste and S its surface area, both constants.

The release rate of nuclide i, $F_i^w(t)$ is derived as

$$F_i^w(t) = R_{wf}(t) I_i(t) S\,, \tag{S4}$$

where $I_i(t)$ is the inventory of nuclide i in mol per kilogram of waste form given by

$$I_i(t) = I_i^0 e^{-\lambda_i t}\,, \tag{S5}$$

where I_i^0 is the initial inventory or nuclide i and λ_i its decay constant in a^{-1}.

2.1.2 Second barrier: buffer form

A buffer of thickness X_B around the waste form constitutes the second barrier to the migration of radionuclides. The flow of nuclides out of the buffer, in $\frac{mol}{a}$, is given by:

$$F_i^B(t) = H\left(t - \tau_i^B\right) F_i^W\left(t - \tau_i^B\right) e^{\left(\lambda_i \tau_i^B\right)}\,. \tag{S6}$$

Since the buffer is supposed to be a purely diffusive barrier, the value of τ_i^B is given by

$$
\begin{aligned}
\tau_i^B &= \tfrac{X_B^2 R_i^B}{4D_B} && [a] \\
R_i^B &= 1 + \tfrac{\rho_B}{\epsilon_B} K_{Di}^B (1-\epsilon_B) && [-] \\
\rho_B &= \text{buffer density} && [\frac{Kg}{m^2}] \\
\epsilon_B &= \text{buffer porosity} && [-] \\
K_{D_i}^B &= \text{buffer sorption constant for nuclide } i && [\frac{m^3}{kg}] \\
D_B &= \text{nuclide independent diffusion coefficient in the buffer} && [\frac{m^2}{a}]
\end{aligned}
\tag{S7}
$$

2.1.3 Third barrier: the geosphere

The nuclear waste is separated from the biosphere by a geological formation (geosphere) of thickness X_G, which both delays and spread the nuclides. The migration into the geosphere is driven by advection (transport by water flow) and dispersion. The flow in $\frac{mol}{a}$ is

$$
F_i^G(t) = H\left(t - \tau_i^B - \tau_i^L\right) H\left(\tau_i^B + \tau_i^H + \tau_i^D - t\right) \frac{\tau_D}{\tau_i^H + \tau_D - \tau_i^L} F_i^B(t') e^{-\lambda_i (t-t')} , \tag{S8}
$$

where

$$
t' = \left(\frac{t - \tau_i^B - \tau_i^L}{\tau_i^H + \tau^D - \tau_i^L} \right) \tau^D + \tau_i^B , \tag{S9}
$$

and τ_i^L, τ_i^H are the upper and lower roots of the following equation of second degree in τ_i^G (in $[m]$):

$$
X_G = 2 \left(\frac{D_G \tau_i^G}{R_i^G} \right)^{\frac{1}{2}} + \frac{V_G \tau_i^G}{R_i^G} \tag{S10}
$$

where

$$
\begin{aligned}
R_i^G &= 1 + \tfrac{\rho_G}{\epsilon_g} (1-\epsilon_G) K_{Di}^G && [-] \\
\rho_G &= \text{geosphere density} && [\frac{kg}{m^3}] \\
\epsilon_G &= \text{geosphere porosity} && [-] \\
K_{Di}^G &= \text{geosphere sorption constant for nuclide } i && [\frac{m^3}{kg}]
\end{aligned}
\tag{S11}
$$

and $D_G = D_G^0 + \alpha_g V_G$, in $\frac{m^2}{a}$, where

$$\begin{aligned} D_G^0 &= \text{diffusion coefficient of nuclide } i \text{ in geosphere} & [\frac{m^2}{a}] \\ \alpha_G &= \text{dispersivity in geosphere} & [m] \\ V_G &= \text{groundwater velocity in geosphere} & [\frac{m}{a}] \end{aligned} \tag{S12}$$

2.1.4 Fourth barrier: biosphere

The model for the biosphere considers that the flow F_i^G coming from the geosphere is entirely intercepted by an abstraction well used for drinking water. The concentration C_i of a given nuclide in the water is given in $\frac{Bq}{m^3}$, where Bq stands for becquerel, the SI unit for radiation:

$$C_i = \frac{F_i^G A_i}{W}\,, \tag{S13}$$

where A_i is the molar specific activity of nuclide i in $\frac{Bq}{mol}$, and W the abstraction rate in $\frac{m^3}{a}$. Thus the resulting dose to humans is simply

$$H_i = C_i W_m D_i\,, \tag{S14}$$

expressed in $\frac{Sv}{a}$, where Sv stands for sievert, the SI unit of dose equivalent describing the biological effect of ionizing radiation. The other terms in the equation are W_m, the water consumption rate by a human drinking the water of the well, in $\frac{m^3}{a}$, and D_i a dose factor converting the ingested becquerels into sieverts.

2.1.5 Uncertain parameters

Tables S1–S2 present the probability distributions used to characterize the uncertain parameters and the constant values of PSACOIN Level 0 respectively.

2.2 The irrigation water witdrawal model

Many Global Hydrological models compute irrigation water withdrawals with variations of the following equation:

$$y = \frac{I_a(ET_c - P)}{E_p}\,, \tag{S15}$$

where y is a scalar representing irrigation water withdrawals [m^3], I_a is the extension of irrigation [m^2], ET_c is the crop evapotranspiration [m], P is the precipitation [m] and E_p is the irrigation efficiency [-].

ET_c is calculated as $ET_c = k_c ET_0$, where ET_0 [m] is the reference crop evapotranspiration (usually grass or alfalfa) and k_c [-] is a coefficient that accounts for the differences between ET_0 and the crop under study (wheat in our case).

Table S1: Probability distributions used to describe the uncertainty in the parameters of the PSACOIN Level 0 model. a = annum.

Input	Unit	Description	Distribution
R^0	$\frac{\text{Kg}}{\text{m}^2}$	Time-invariant leach rate	$Logunif(10^{-2.57}, 10^{1.11})$
X_B	m	Thickness of buffer	$\mathcal{U}(0.5, 5)$
K_D^B	$\frac{\text{m}^3}{\text{kg}}$	Buffer sorption constant	$Lognorm(-2.38, 0.143)$
K_D^G	$\frac{\text{m}^3}{\text{kg}}$	Geosphere soprtion constant	$Lognorm(-3.38, 0.3)$
D_G^0	$\frac{\text{m}^2}{\text{a}}$	Diffusion coefficient	$\mathcal{N}(0.04, 0.001)$
X_G	m	Thickness of geosphere	$\mathcal{U}(10^3, 10^4)$
α_G	m	Dispersivity in geosphere	$Logunif(10^{0.3}, 10^{2.3})$
V_G	$\frac{\text{m}}{\text{a}}$	Groundwater velocity in geosphere	$Logunif(10^{-3}, 10^{-1})$
W	$\frac{\text{m}^3}{\text{a}}$	Abstraction rate	$\mathcal{U}(5 \times 10^5, 5 \times 10^6)$
W_m	$\frac{\text{m}^3}{\text{a}}$	Human water consumption rate	$\mathcal{U}(0.7, 0.9)$

In the paper we consider two different equations for ET_0, the Priestley-Taylor and the FAO-56 Penman-Monteith [42]. The former reads as

$$ET_0 = \alpha \frac{\Delta A}{\Delta + \gamma}, \quad \text{(S16)}$$

whereas the latter reads as

$$ET_0 = \frac{0.408\Delta A + \gamma \frac{900}{T_a + 273} wv}{\Delta + \gamma(1 + 0.34w)}, \quad \text{(S17)}$$

where A is the net radiation minus the soil heat flux (MJ m^{-2} d^{-1}), Δ the gradient of saturated vapour pressure (kPa ºC^{-1}), γ the psychometric constant (kPa ºC^{-1}), α the Priestley-Taylor constant, T_a the mean daily air temperature at 2m (ºC), w the average daily wind speed at 2m (m s^{-1}) and v the vapor pressure deficit (kPa). See Allen [70] for an explanation of the constants.

2.2.1 Uncertain parameters

See Puy et al. [41, 71] for an explanation of the uncertainties involved in the calculation of irrigation water withdrawals, including the selection of the probability distributions used in this paper (Table S3).

2.3 The epidemiological models

We use the Susceptible-Infected-Recovered [SIR(S)] models by Saad-Roy et al. [45] (Equations S18–S19) and by Saad-Roy et al. [46] (Equation S20). See Tables S4–S5 for a description of the parameters and coefficients, and Saad-Roy et al. [45, 46] for a full explanation of the models' dynamics.

The most simple SIR(S) reads as

Table S2: Constants of the PSACOIN Level 0 model. a = annum.

Constant	Unit	Description	Value
I_0	$\frac{\text{mol}}{\text{Kg}}$	Initial inventory of radionuclide	$2.035 \times 10{-}5$
λ	a^{-1}	Decay constant of radionuclide	$1.07 \times 10{-}5$
S	m^2	Surface area of radionuclide	1.2×10^6
Q	Kg	Initial amount of waste	2×10^8
ρ_B	$\frac{\text{Kg}}{\text{m}^2}$	Buffer density	1.85×10^3
ϵ_B	-	Buffer porosity	0.099
D_B	$\frac{\text{m}^2}{\text{a}}$	Diffusion coefficient in buffer	0.03
ρ_G	$\frac{\text{Kg}}{\text{m}^3}$	Geosphere density	1.85×10^3
ϵ_G	-	Geosphere porosity	0.3
A	$\frac{\text{Bq}}{\text{mol}}$	Molar specific activity	2.04×10^{11}
D	$\frac{\text{Sv}}{\text{Bq}}$	Dose factor conversion	2.3×10^{-9}

$$
\begin{aligned}
\frac{dS_P}{dt} &= \mu N - \beta S_P \frac{I_P + \alpha I_S}{N} - \mu S_P \\
\frac{dI_P}{dt} &= \beta S_P \frac{I_P + \alpha I_S}{N} - (\gamma + \mu) I_P \\
\frac{dR}{dt} &= \gamma (I_P + I_S) - (\delta + \mu) R \\
\frac{dS_S}{dt} &= \delta R - \epsilon \beta S_S \frac{I_P + \alpha I_S}{N} - \mu S_S \\
\frac{dI_S}{dt} &= \epsilon \beta S_S \frac{I_P + \alpha I_S}{N} - (\gamma + \mu) I_S
\end{aligned}
\tag{S18}
$$

where S_P denotes fully susceptible individuals, I_P denotes individuals with primary infection that transmit at rate β, R denotes fully immune individuals (as a result of recovery), S_S denotes individuals whose immunity has waned at rate δ and are again susceptible to infection, and I_S denotes individuals with secondary infection.

The SIR(S) with a vaccination term reads as

Table S3: Summary of the uncertainty in the parameters of the irrigation water withdrawal model.

Input	Description	Distribution
Δ	Vapour pressure	$\mathcal{U}(0.0796, 0.0804)$
γ	Psychrometric constant	$\mathcal{U}(0.065, 0.066)$
A	Net radiation minus soil heat flux	$\mathcal{U}(297.55, 402.448)$
T_a	Mean air temperature	$\mathcal{U}(9.9, 10.1)$
w	Wind speed	$\mathcal{U}(2.67, 2.95)$
v	Vapor deficit	$\mathcal{U}(0.26, 0.29)$
k_c	Crop coefficient	$\mathcal{U}(0.45, 1.14)$
I_a	Irrigated area	$\mathcal{U}(42.9, 144.5)$
E_a	Field application efficiency	$\mathcal{U}(0.49, 0.88)$
E_c	Conveyance efficiency	$\mathcal{U}(0.64, 0.96)$
M_f	Management factor	$\mathcal{U}(0.5, 0.97)$
P	Precipitation	$\mathcal{U}(0, 0.1)$

$$\begin{aligned}
\frac{dS_P}{dt} &= \mu N - \beta S_P \frac{I_P + \alpha I_S}{N} - \mu S_P - s_{vax}\nu S_P \\
\frac{dI_P}{dt} &= \beta S_P \frac{I_P + \alpha I_S}{N} - (\gamma + \mu) I_P \\
\frac{dR}{dt} &= \gamma (I_P + I_S) - (\delta + \mu) R \\
\frac{dS_S}{dt} &= \delta R - \epsilon \beta S_S \frac{I_P + \alpha I_S}{N} - \mu S_S + \delta_{vax} V - s_{vax}\nu S_S \\
\frac{dI_S}{dt} &= \epsilon \beta S_S \frac{I_P + \alpha I_S}{N} - (\gamma + \mu) I_S \\
\frac{dV}{dt} &= s_{vax}\nu (S_P + S_S) - \delta_{vax} V - \mu V
\end{aligned} \tag{S19}$$

where V denotes vaccinated individuals.

The SIR(S) extended with different vaccination strategies reads as

$$
\begin{aligned}
\frac{dS_P}{dt} &= \mu - \beta S_P(I_P + \alpha I_S + \alpha V I_V + \alpha_1 I_{S_1} + \alpha_2 I_{S_2}) - \mu S_P - s_{vax}\nu S_P \\
\frac{dI_P}{dt} &= \beta S_P(I_P + \alpha I_S + \alpha_V I_V + \alpha_1 I_{S_1} + \alpha_2 I_{S_2}) - (\gamma + \mu) I_P \\
\frac{dR}{dt} &= \gamma(I_P + I_S + I_V + I_{S_1} + I_{S_2}) - (\delta + \mu) R \\
\frac{dS_S}{dt} &= \delta R - \epsilon \beta S_S(I_P + \alpha I_S + \alpha_V I_V + \alpha_1 I_{S_1} + \alpha_2 I_{S_2}) - \mu S_S - s_{vax}\nu S_S \\
\frac{dI_S}{dt} &= \epsilon \beta S_S(I_P + \alpha I_S + \alpha_V I_V + \alpha_1 I_{S_1} + \alpha_2 I_{S_2}) - (\gamma + \mu) I_S \\
\frac{dV_1}{dt} &= s_{vax}\nu(S_P + dS_S) - \epsilon_{V_1}\beta V_1(I_P + \alpha I_S + \alpha_V I_V + \alpha_1 I_{S_1} + \alpha_2 I_{S_2}) - (\omega + \rho_1 + \mu) V_1 \\
\frac{dV_2}{dt} &= (1-d) s_{vax}\nu S_S + \omega V_1 - \epsilon_{V_2}\beta V_2(I_P + \alpha I_S + \alpha_V I_V + \alpha_1 I_{S_1} + \alpha_2 I_{S_2}) - (\rho_2 + \mu) V_2 \\
\frac{dI_V}{dt} &= \beta(\epsilon_{V_1} V_1 + \epsilon_{V_2} V_2)(I_P + \alpha I_S + \alpha_V I_V + \alpha_1 I_{S_1} + \alpha_2 I_{S_2}) - (\gamma + \mu) I_V \\
\frac{dS_{S_1}}{dt} &= \rho_1 V_1 - \epsilon_1 \beta S_{S_1}(I_P + \alpha I_S + \alpha_V I_V + \alpha_1 I_{S_1} + \alpha_2 I_{S_2}) - \mu S_{S_1} \\
\frac{dS_{S_2}}{dt} &= \rho_2 V_2 - \epsilon_2 \beta S_{S_2}(I_P + \alpha I_S + \alpha_V I_V + \alpha_1 I_{S_1} + \alpha_2 I_{S_2}) - \mu S_{S_2} \\
\frac{dI_{S_1}}{dt} &= \epsilon_1 \beta S_{S_1}(I_P + \alpha I_S + \alpha_V I_V + \alpha_1 I_{S_1} + \alpha_2 I_{S_2}) - (\gamma + \mu) I_{S_1} \\
\frac{dI_{S_2}}{dt} &= \epsilon_2 \beta S_{S_2}(I_P + \alpha I_S + \alpha_V I_V + \alpha_1 I_{S_1} + \alpha_2 I_{S_2}) - (\gamma + \mu) I_{S_2}
\end{aligned}
\tag{S20}
$$

where V_i denotes individuals vaccinated with i doses, S_{S_i} denotes individuals whose complete i-dose immunity has waned and are susceptible again, I_{S_i} denotes individuals who were in S_{S_i} and have now been infected again, and I_V denotes individuals for whom the vaccine did not prevent infection.

2.3.1 Uncertain parameters

Tables S4–S5 respectively present the probability distributions used to characterize the uncertain parameters and the constant values of the epidemiological models.

Table S4: Probability distributions used to describe the uncertainty in the parameters of the epidemiological models, selected from Saad-Roy et al. [45, 46].

Input	Description	Distribution
ϵ	Reduction in susceptibility to secondary infections relative to primary ones	$\mathcal{U}(0.4, 1)$
$\alpha, \alpha_1, \alpha_2, \alpha_V$	Reduction in the infectiousness of secondary infections relative to primary ones	$\mathcal{U}(0.8, 1)$
ν	Fraction of the fully and partially susceptible populations vaccinated each week	$\mathcal{U}(0.001, 0.009)$
t_{vax}	Time at which vaccination is introduced	$\mathcal{U}(48, 78)$

Table S5: Constants of the epidemiological models by Saad-Roy et al. [45, 46].

Constant	Description	Value
γ	Recovery rate primary / secondary infections	7 / 5
δ	Wane rate of full immunity from infection	1 / 52
μ	Birth rate to enter the susceptible class S_P	1 / (50 $\times$ 52)
ϵ_{V_1}	First level of immune protection	0.1
ϵ_{V_2}	Second level of immune protection	0.05
ω	Interdose period	0
ρ_1	Waning rate of vaccinal immunity 1	0
ρ_2	Waning rate of vaccinal immunity 2	0
δ_{vax}	Rate at which vaccinal immunity is lost	1
ϵ_1	Effect of vaccine 1	0.7
ϵ_2	Effect of vaccine 2	0.7
d	Fraction of previously infected partially susceptible individuals (SS) for whom one dose of the vaccine gives equivalent immunity to two doses for fully susceptible individuals	0.5

REFERENCES AND NOTES

1. E. F. Wood, J. K. Roundy, T. J. Troy, L. P. H. van Beek, M. F. P. Bierkens, E. Blyth, A. de Roo, P. Döll, M. Ek, J. Famiglietti, D. Gochis, N. van de Giesen, P. Houser, P. R. Jaffé, S. Kollet, B. Lehner, D. P. Lettenmaier, C. Peters-Lidard, M. Sivapalan, J. Sheffield, A. Wade, P. Whitehead, Hyperresolution global land surface modeling: Meeting a grand challenge for monitoring earth's terrestrial water. *Water Resour. Res.* **47**, W05301 (2011).

2. T. Palmer, Climate forecasting: Build high-resolution global climate models. *Nature* **515**, 338–339 (2014).

3. M. F. P. Bierkens, V. A. Bell, P. Burek, N. Chaney, L. E. Condon, C. H. David, A. de Roo, P. Döll, N. Drost, J. S. Famiglietti, M. Flörke, D. J. Gochis, P. Houser, R. Hut, J. Keune, S. Kollet, R. M. Maxwell, J. T. Reager, L. Samaniego, E. Sudicky, E. H. Sutanudjaja, N. van de Giesen, H. Winsemius, E. F. Wood, Hyper-resolution global hydrological modelling: What is next?: "Everywhere and locally relevant". *Hydrol. Process.* **29**, 310–320 (2015).

4. G. E. Moore, Cramming more components onto integrated circuits. *Electronics* **38**, 114 (1965).

5. R. H. Dennard, F. H. Gaensslen, H.-N. Yu, V. L. Rideout, E. Bassous, A. R. LeBlanc, Design of ion-implanted mosfet's with very small physical dimensions. *IEEE J. Solid State Circuits* **9**, 256–268 (1974).

6. Z. Hausfather, H. F. Drake, T. Abbott, G. A. Schmidt, Evaluating the performance of past climate model projections. *Geophys. Res. Lett.* **47**, e2019GL085378 (2020).

7. M. C. Sarofim, J. B. Smith, A. St. Juliana, C. Hartin, Improving reduced complexity model assessment and usability. *Nat. Clim. Chang.* **11**, 1–3 (2021).

8. S. Manabe, Climate and the ocean circulation I. The atmospheric circulation and the hydrology of the Earth's surface. *Mon. Weather Rev.* **97**, 739–774 (1969).

9. M. F. P. Bierkens, Global hydrology 2015: State, trends, and directions. *Water Resour. Res.* **51**, 4923–4947 (2015).

10. W. O. Kermack, A. G. McKendrick, Contributions to the mathematical theory of epidemics. III.—Further studies of the problem of endemicity. *Proc. R. Soc. Lond.* **141**, 94–122 (1933).

11. N. M. Ferguson, D. Laydon, G. Nedjati Gilani, N. Imai, K. Ainslie, M. Baguelin, S. Bhatia, A. Boonyasiri, Z. Cucunuba, G. Cuomo-Dannenburg, A. Dighe, I. Dorigatti, H. Fu, K. Gaythorpe, W. Green, A. Hamlet, W. Hinsley, L. C. Okell, S. Van Elsland, H. Thompson, R. Verity, E. Volz, H. Wang, Y. Wang, P. G. T. Walker, C. Walters, P. Winskill, C. Whittaker, C. A. Donnelly, S. Riley, A. C. Ghani, Report 9: Impact of non-pharmaceutical interventions (NPIs) to reduce COVID-19 mortality and healthcare demand, *Tech. Rep. March* (Imperial College COVID-19 Response Team, 2020).

12. J. C. Refsgaard, J. van der Sluijs, J. Brown, P. van der Keur, A framework for dealing with uncertainty due to model structure error. *Adv. Water Resour.* **29**, 1586–1597 (2006).

13. H. Akaike, Akaike's Information Criterion, in *International Encyclopedia of Statistical Science*, M. Lovric, Ed. (Springer Berlin Heidelberg, 2011), p. 25.

14. G. Schwarz, Estimating the dimension of a model. *Ann. Stat.* **6**, 461–464 (1978).

15. A. Saltelli, G. Bammer, I. Bruno, E. Charters, M. Di Fiore, E. Didier, W. Nelson Espeland, J. Kay, S. Lo Piano, D. Mayo, R. Pielke Jr, T. Portaluri, T. M. Porter, A. Puy, I. Rafols, J. R. Ravetz, E. Reinert, D. Sarewitz, P. B. Stark, A. Stirling, J. van der Sluijs, P. Vineis, Five ways to ensure that models serve society: A manifesto. *Nature* **582**, 482–484 (2020).

16. O. H. Pilkey, L. Pilkey-Jarvis, *Useless Arithmetic: Why Environmental Scientists Can't Predict the Future* (Columbia Univ. Press, 2009).

17. R. E. Caflisch, W. Morokoff, A. Owen, Valuation of mortgage-backed securities using Brownian bridges to reduce effective dimension. *J. Comput. Finance* **1**, 27–46 (1997).

18. X. Wang, K. T. Fang, The effective dimension and quasi-Monte Carlo integration. *J. Complex.* **19**, 101–124 (2003).

19. S. Kucherenko, B. Feil, N. Shah, W. Mauntz, The identification of model effective dimensions using global sensitivity analysis. *Reliab. Eng. Syst. Saf.* **96**, 440–449 (2011).

20. A. Saltelli, K. Aleksankina, W. Becker, P. Fennell, F. Ferretti, N. Holst, S. Li, Q. Wu, Why so many published sensitivity analyses are false: A systematic review of sensitivity analysis practices. *Environ. Model. Software* **114**, 29–39 (2019).

21. R. J. Brooks, A. M. Tobias, Choosing the best model: Level of detail, complexity, and model performance. *Math. Comput. Model.* **24**, 1–14 (1996).

22. H. A. Simon, The architecture of complexity. *Proc. Am. Philos. Soc.* **106**, 467–482 (1962).

23. S. M. Manson, Simplifying complexity: A review of complexity theory. *Geoforum* **32**, 405–414 (2001).

24. H. M. Regan, M. Colyvan, M. A. Burgman, A taxonomy and treatment of uncertainty for ecology and conservation biology. *Ecol. Appl.* **12**, 618–628 (2002).

25. I. M. Sobol', Sensitivity estimates for nonlinear mathematical models. *Math. Model. Comput. Exp.* **4**, 407–414 (1993).

26. T. Homma, A. Saltelli, Importance measures in global sensitivity analysis of nonlinear models. *Reliab. Eng. Syst. Saf.* **52**, 1–17 (1996).

27. V. Pareto, *Manuale di Economia Politica* (Societa Editrice, 1906), vol 13.

28. G. E. P. Box, R. D. Meyer, An analysis for unreplicated fractional factorials. *Dent. Tech.* **28**, 11–18 (1986).

29. W. Becker, Metafunctions for benchmarking in sensitivity analysis. *Reliab. Eng. Syst. Saf.* **204**, 107189 (2020).

30. B. Edmonds, "Syntactic measures of complexity," thesis, University of Manchester (1999).

31. A. Puy, W. Becker, S. Lo Piano, A. Saltelli, A comprehensive comparison of total-order estimators for global sensitivity analysis. *Int. J. Uncertain. Quantif.* **12**, 1–18 (2022).

32. R. L. Wilby, S. Dessai, Robust adaptation to climate change. *Weather* **65**, 180–185 (2010).

33. M. Maslin, Cascading uncertainty in climate change models and its implications for policy. *Geogr. J.* **179**, 264–271 (2013).

34. F. Pappenberger, K. J. Beven, N. M. Hunter, P. D. Bates, B. T. Gouweleeuw, J. Thielen, A. P. de Roo, Cascading model uncertainty from medium range weather forecasts (10 days) through a rainfall-runoff model to flood inundation predictions within the European Flood Forecasting System (EFFS). *Hydrol. Earth Syst. Sci.* **9**, 381–393 (2005).

35. P. Magal, S. Ruan, Susceptible-infectious-recovered models revisited: From the individual level to the population level. *Math. Biosci.* **250**, 26–40 (2014).

36. B. W. Goodwin, A. E. C. L. Canada, A. Saltelli, PSACOIN Level 0 Intercomparison, *Tech. Rep.* (Nuclear Energy Agency, Organisation for Economic Co-operation and Development, Paris, 1987).

37. D. Metlay, From tin roof to torn wet blanket: Predicting and observing ground water movement at a proposed nuclear waste site, in *Prediction: Science, Decision Making, and the Future of Nature*, D. Sarewitz, R. A. Pielke Jr., R. Byerly Jr., Eds. (Island Press, 2000).

38. D. Sarewitz, "Of cold mice and isotopes or should we do less science?," in *Exploring Relations Between Academic Research, Higher Education and Science Policy. Summer School in Higher Education Research and Science Studies* (Universität Bonn, 2018), pp. 1–8.

39. M. Brugnach, Process level sensitivity analysis for complex ecological models. *Ecol. Model.* **187**, 99–120 (2005).

40. A. Puy, R. Sheikholeslami, H. V. Gupta, J. W. Hall, B. Lankford, S. Lo Piano, J. Meier, F. Pappenberger, A. Porporato, G. Vico, A. Saltelli, The delusive accuracy of global irrigation water withdrawal estimates. *Nat. Commun.* **13**, 3183 (2022).

41. J. Lu, G. Sun, S. G. McNulty, D. M. Amatya, A comparison of six potential evapotranspiration methods for regional use in the southeastern United States. *J. Am. Water Resour. Assoc.* **41**, 621–633 (2005).

42. T. A. McMahon, M. C. Peel, L. Lowe, R. Srikanthan, T. R. McVicar, Estimating actual, potential, reference crop and pan evaporation using standard meteorological data: A pragmatic synthesis. *Hydrol. Earth Syst. Sci.* **17**, 1331–1363 (2013).

43. A. Puy, S. Lo Piano, A. Saltelli, Current models underestimate future irrigated areas. *Geophys. Res. Lett.* **47**, e2020GL087360 (2020).

44. V. E. Pitzer, C. Viboud, L. Simonsen, C. Steiner, C. A. Panozzo, W. J. Alonso, M. A. Miller, R. I. Glass, J. W. Glasser, U. D. Parashar, B. T. Grenfell, Demographic variability, vaccination, and the spatiotemporal dynamics of rotavirus epidemics. *Science* **325**, 290–294 (2009).

45. C. M. Saad-Roy, C. E. Wagner, R. E. Baker, S. E. Morris, J. Farrar, A. L. Graham, S. A. Levin, M. J. Mina, C. J. E. Metcalf, B. T. Grenfell, Immune life history, vaccination, and the dynamics of SARS-CoV-2 over the next 5 years. *Science* **370**, 811–818 (2020).

46. C. M. Saad-Roy, S. E. Morris, C. J. E. Metcalf, M. J. Mina, R. E. Baker, J. Farrar, E. C. Holmes, O. G. Pybus, A. L. Graham, S. A. Levin, B. T. Grenfell, C. E. Wagner, Epidemiological and evolutionary considerations of SARS-CoV-2 vaccine dosing regimes. *Science* **372**, 363–370 (2021).

47. P. C. Stern, J. H. Perkins, R. E. Sparks, R. A. Knox, The challenge of climate-change neoskepticism. *Science* **353**, 653–654 (2016).

48. L. Maxim, J. P. van der Sluijs, Uncertainty: Cause or effect of stakeholders' debates?. *Sci. Total Environ.* **376**, 1–17 (2007).

49. S. Funtowicz, J. R. Ravetz, *Uncertainty and Quality in Science for Policy* (Kluwer Academic Publishers, 1990).

50. R. V. O'Neill, Error analysis of ecological models, in *Radionuclides in Ecosystems. Proceedings of the Third National Symposium on Radioecology, May 10–12, 1971, Oak Ridge, Tennessee*, D. J. Nelson, Ed. (Springfield, 1971).

51. L. A. Zadeh, Outline of a new approach to the analysis of complex systems and decision processes. *IEEE Trans. Syst. Man Cybern.* **SMC-3**, 28–44 (1973).

52. S. Geman, E. Bienenstock, R. Doursat, Neural networks and the bias/variance dilemma. *Neural Comput.* **4**, 1–58 (1992).

53. G. Gigerenzer, J. N. Marewski, Surrogate science: The idol of a universal method for scientific inference. *J. Manag.* **41**, 421–440 (2015).

54. W. E. Walker, V. A. Marchau, D. Swanson, Addressing deep uncertainty using adaptive policies: Introduction to section 2. *Technol. Forecast. Soc. Change* **77**, 917–923 (2010).

55. S. Robertson, Transparency, trust, and integrated assessment models: An ethical consideration for the intergovernmental panel on climate change. *Wiley Interdiscip. Rev. Clim. Chang.* **12** e679 (2021).

56. L. van Beek, J. Oomen, M. Hajer, P. Pelzer, D. van Vuuren, Navigating the political: An analysis of political calibration of integrated assessment modelling in light of the 1.5 °C goal. *Environ. Sci. Policy* **133**, 193–202 (2022).

57. A. Saltelli, A. G. Pereira, J. P. van der Sluijs, S. O. Funtowicz, What do I make of your latinorum? Sensitivity auditing of mathematical modelling. *Int. J. Innov. Pol.* **9**, 213–234 (2013).

58. E. S. Quade, Pitfalls in formulation and modeling, in *Pitfalls of Analysis*, G. Majone, E. S. Quade, Eds. (John Wiley & Sons, 1980), pp. 23–43.

59. B. Duignan, Occam's razor, in *Encyclopedia Britannica* (2021), [accesssed 24 August 2022]; www.britannica.com/topic/Occams-razor.

60. P. Döll, S. Siebert, Global modeling of irrigation water requirements. *Water Resour. Res.* **38**, 8–1–8–10 (2002).

61. E. H. Sutanudjaja, L. P. H. van Beek, N. Drost, I. E. M. de Graaf, K. de Jong, S. Peßenteiner, M. W. Straatsma, Y. Wada, N. Wanders, D. Wisser, M. F. P. Bierkens, PCR-GLOBWB 2: A 5 arcmin global hydrological and water resources model. *Geosci. Model Dev.* **11**, 2429–2453 (2018).

62. A. Puy, E. Borgonovo, S. Lo Piano, S. A. Levin, A. Saltelli, Irrigated areas drive irrigation water withdrawals. *Nat. Commun.* **12**, 4525 (2021).

63. A. Saltelli, M. Ratto, T. Andres, F. Campolongo, J. Cariboni, D. Gatelli, M. Saisana, S. Tarantola, *Global Sensitivity Analysis. The Primer* (John Wiley & Sons Ltd, 2008).

64. W. S. Parker, Ensemble modeling, uncertainty and robust predictions. *Wiley Interdiscip. Rev. Clim. Chang.* **4**, 213–223 (2013).

65. I. M. Sobol', On the distribution of points in a cube and the approximate evaluation of integrals. *USSR Comput. Math. Math. Phys.* **7**, 86–112 (1967).

66. I. Azzini, T. Mara, R. Rosati, Comparison of two sets of Monte Carlo estimators of Sobol' indices. *Environ. Model. Software* **144**, 105167 (2021).

67. A. Puy, S. Lo Piano, A. Saltelli, S. A. Levin, sensobol: An R package to compute variance-based sensitivity indices. *J. Stat. Softw.* **102**, 1–37 (2022).

68. I. M. Sobol', On quasi-Monte Carlo integrations. *Math. Comput. Simul.* **47**, 103–112 (1998).

69. A. Puy, R code for "Models with higher effective dimensions tend to produce more uncertain estimates". *Zenodo* (2021); https://doi.org/10.5281/zenodo.5658383.

70. R. G. Allen, L. S. Pereira, D. Raes, M. Smith, Crop evapotranspiration: Guidelines for computing crop water requirements. *Irrig. Drain.* **300**, 300 (1998).

71. A. Puy, R. Sheikholeslami, H. V. Gupta, J. W. Hall, B. Lankford, S. Lo Piano, J. Meier, F. Pappenberger, A. Porporato, G. Vico, A. Saltelli, Supplementary Materials for The delusive accuracy of global irrigation water withdrawal estimates. *Zenodo* (2021); https://doi.org/10.5281/zenodo.5528844.
